# Using the child behavior checklist to determine associations between psychosocial aspects and TMD-related pain in children and adolescents

**DOI:** 10.1186/s10194-018-0915-6

**Published:** 2018-09-21

**Authors:** Amal Al-Khotani, Mattias Gjelset, Aron Naimi-Akbar, Britt Hedenberg-Magnusson, Malin Ernberg, Nikolaos Christidis

**Affiliations:** 1 Division of Oral Diagnostics and Rehabilitation, Department of Dental Medicine, Karolinska Institutet, Box 4064, SE-141 04 Huddinge, Sweden; 2 East Jeddah Hospital, Ministry of health, Jeddah, Saudi Arabia; 3 Scandinavian Center for Orofacial Neurosciences (SCON), Huddinge, Sweden; 4 Oral and maxillofacial surgery, Department of Dental Medicine, Karolinska Institutet, Huddinge, Sweden; 5 Department of Clinical Oral Physiology at the Eastman Institute, Stockholm Public Dental Health, Stockholm, Sweden

**Keywords:** Pain, Psychology, Children, Adolescents, Child behaviour checklist

## Abstract

**Background:**

Since children and adolescents are frequently experiencing emotional and behavioral consequences due to pain, their parents should be aware of this emotional and behavioral status. Therefore, the aim of this study was to analyze and describe the parents’ reports of the emotional and behavioral status of children and adolescents with different types of temporomandibular disorders using the Child Behavior Checklist.

**Methods:**

This Cross-sectional study comprises of 386 randomly selected children and adolescents that ages between 10 and 18 years in Jeddah. One day prior the clinical examination according to Research Diagnostic Criteria for temporomandibular disorders (TMD) Axis I and II, Arabic version of the Child Behavior Checklist scale was distributed to the parents of participant. According to the diagnosis, the participants were divided into three groups; non-TMD group, TMD-pain group, and TMD-painfree group.

**Results:**

In regard to internalizing problems, the parents to the children and adolescents in the TMD-pain group rated a higher frequency of anxiety, depression and somatic complaints in their children than the parents of children in the non-TMD group (*p* < 0.05). Only one significant association regarding the externalizing problems was found for the aggressive behavior in the TMD-pain group.

**Conclusion:**

The parents rated that their children with TMD-pain suffer from emotional, somatic and aggressive behavior to a higher degree than healthy control subjects. Also, the parents believed that TMD-pain influenced their children’s physical activities but not social activities.

## Background

During the last decades, pain among children and adolescents has been recognized as a significant health problem. As the practice of pediatric pain has progressed a lot, also the impact that chronic pain has on the children’s daily living has been documented. This includes limitations in both social and physical functioning as well as their family’s well-being [1]. It has been shown that children and adolescents are frequently experiencing emotional and behavioral consequences due to pain [2]. Therefore, it is of great importance that the emotional and behavioral status of the children is not just evaluated but also known by their parents.

One way to assess parents’ knowledge regarding their children’s emotional and behavioral status is by using questionnaires handed to the parents. One of those is the Child Behavior Checklist (CBCL) [3] that has been used to describe the psychosocial status of nurture children and adolescents. It should be completed by the child’s parent or child’s caregiver who has had the child for a period equal to or more than six months. CBCL measures not only the emotional, behavior and physical problems in school-age children from 6 to 18 years old, but it also reports the child’s social competence such as social and peer relationships, as well as family relationships [3]. CBCL has been used in previous studies to explore the emotional and behavior problems in children and adolescents and to correlate these psychometric measures to different pain conditions such as juvenile chronic arthritis (JIA), pediatric cancer, and hematological conditions (4, 5). In one study, CBCL showed that children with cancer and hematological condition suffered from internalizing symptoms such as anxiety, depression and somatic problems [4], while another study reported no association between pain and child psychosocial functioning [5]. Other studies have also used CBCL to assess the psychosocial profile of children with sleep disorders, headache, abdominal pain and irritable bowel syndrome. Those studies showed that children reported at least one emotional and/or behavioral disorder [6–8]. Furthermore, CBCL was used to evaluate the mental health of children living with a mother suffering from chronic pain [9].

Temporomandibular disorders (TMD) in children and adolescents seem to be a more significant problem than previously believed with a prevalence reaching up to 27.2% [10]. Earlier studies have shown prevalences that range between 4.2% and 27% [10, 11]. Recent studies have used different psychometric measures in children and adolescents suffering from TMD [2, 12–14]. These methodological differences between studies are present since it until recently was no suitable instrument measuring the TMD-associated problems for youngsters suffering from emotional and somatic pain. Similar to other conditions, TMD is accompanied by comorbid and somatization disorders (psychological suffering that felt like a real somatic pain) [15]. Also, in psychological studies, a comorbidity with psychiatric conditions and psychological distress has been shown to significantly and negatively modify the outcome of patients with chronic conditions like headache, making it a reliable predictor of suicidal risk [16]. Therefore, there is a great need to use a proper psychometric approach to analyze all possible TMD-related problems. Most of the studies have used the questionnaires from the Research Diagnostic Criteria for Temporomandibular Disorders (RDC/TMD) Axis II [13, 14], which are validated for adults. Other studies have used specially constructed questionnaires to ask the parents about their children’s emotional status when they complain of TMD [12]. Further, just a few studies have used the Youth self-report (YSR) [17], which is a “child-rating scale” analogous to CBCL, to evaluate emotional and behavioral functioning in children and adolescents [2, 15]. A recent study from our research group used YSR to assess the emotional, behavior and somatic functioning among children and adolescents with various TMD condition (2). The same study found a significant association between TMD-pain and anxiety, depression, somatic problems, aggressive behavior as well as thought problems. Until now, the emotional effects on children with different TMD-pain conditions have not been assessed using the “parent- rating scale”, i.e. the CBCL.

Taken together, we hypothesized that psychosocial problems in children and adolescents are associated with a diagnosis of TMD with pain (TMD-pain) but not with a diagnosis of TMD without pain (TMD-pain-free) when CBCL is used. Therefore, the purpose of the present study was to analyze and describe the parents’ reports of the emotional and behavioral status of children and adolescents with different types of temporomandibular disorders (TMD) using the Child Behavior Checklist (CBCL).

## Methods

This study is part of a larger project from our research group. This cross-sectional study followed the guidelines of the Declaration of Helsinki and was approved by the local ethics committee at the Department of Medical Study and Research, Ministry of Health, Jeddah, Saudi Arabia. Before inclusion, all participants or their parents gave both verbal and written consent, after receiving written information. An extended verbal explanation was further provided upon request.

**Fig. 1:**
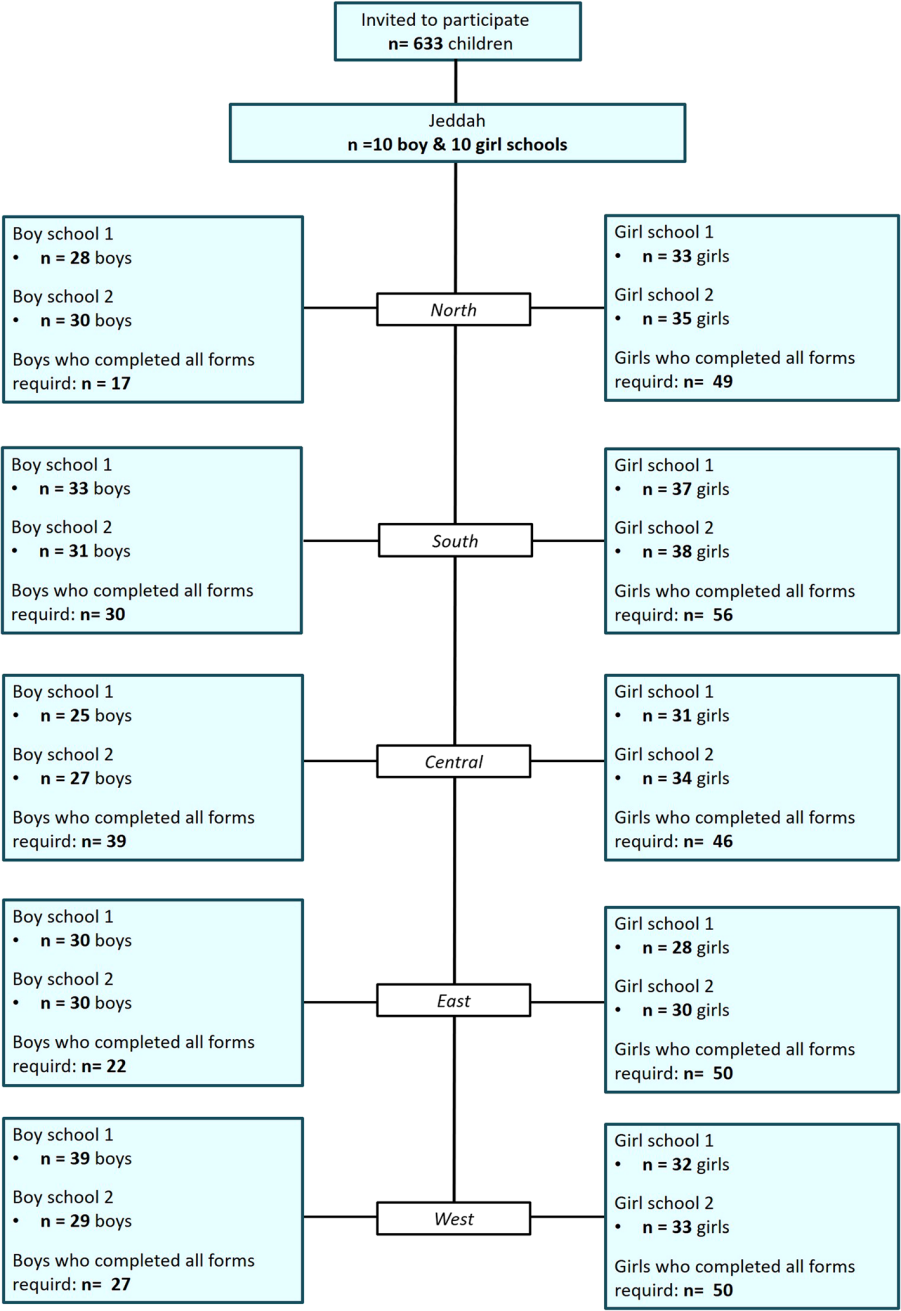
Flowchart diagram showing the distribution of 386 children and adolescents, Jeddah, Saudi Arabia

In the current study, a total of 633 children and adolescents of both sexes, aged between 10 and 18 years were asked to participate, 509 of them agreed to participate. The sample consisted of children among the general population of Jeddah; a major and cosmopolitan city in Saudi Arabia. A total of 386 parents completed the questionnaire. The flow-chart (Fig. 1) illustrates not just the participation-rate from the different areas of Jeddah, but also how many completed forms were handed in, among boys and girls separately. However, we do not have the information on why the forms were not handed in, neither why some children did not participate at the clinical examination.

### Study setting

The city of Jeddah was divided into five regions (North, South, East, West, and Central). Because the education in Saudi Arabia is based on single-sex schools: two schools with boys and two schools with girls were randomly selected from the predefined list of schools from each region as grouped by the ministry of education, with children aged 10–18. The randomization was performed by the principal investigator (NC), who did not participate in the data collection, with an internet-based application (www.randomization.com) [10]. One class from each school was chosen, using stratified selction based on age for the randomization in order to get the most representative and homogenous matrerial as possible. This randomization was also performed by NC, with an average of 28 pupils. A dental assistant who did not participate in the project drew the school classes’ titles from a bucket by using simple sampling method. One day before visiting each school, a sealed envelope containing proper information about the study, questions regarding demographic data (including ethnic, socioeconomic background information, and medical history), the CBCL questionnaires, as well as the consent form, was distributed to the parents through their children. Due to cultural considerations, boys were invited to be examined in the dental clinic of the primary health care center in each region, while girls’ examination was performed in girls’ school at the school nurse’s room.

On the day of the examination, all envelopes that were sent to the parents were collected, followed by a verbal explanation about the aim of the study by one examiner (AA-K). Each participant was asked two validated questions about the presence of orofacial pain (TMD-pain) [11]; 1) “Do you have pain in the temple, face, temporomandibular joint, or jaws once a week or more?” 2) “Do you have pain when you open your mouth wide or chew once a week or more?”. The clinical examination was performed according to the RDC/TMD (Axis I) protocol by one examiner (A A-K), who was trained by an orofacial pain specialist (ME; calibrated to a gold-standard examiner (Thomas List)). The RDC/TMD protocol was chosen since it is reliable as an examination protocol for children with TMD [11, 18], while the newer DC/TMD [19] was not used on children at that time.

#### Measures

##### Research diagnostic criteria for temporomandibular disorders Axis I

The RDC/TMD is a standardized dual diagnostic method for TMD [20]. It was developed to increase the strength and reliability of the results of multicenter projects, e.g. when various specialties from different countries collaborate, but also to enable comparison of results between studies. This diagnostic tool consists of Axis I, a clinical examination, and Axis II, a biobehavioral questionnaire. In Axis I, the TMJ and orofacial muscles are examined to diagnose the presence of one of the following clinical clusters: muscle disorders, internal derangement, or degenerative joint disorders. In the Axis II, the biobehavioral section is divided into four primary domains: the GCPS, the Jaw Disability Checklist (JDC), the Depression and Non-specific Physical Symptoms scales (SCL-90-R), and patient characteristics. Axis I has been shown to be reliable in children and adolescents [11]. However, the SCL-90-R of the Axis II has not been validated for children and adolescents younger than 13 years of age [21]. Therefore, the SCL-90-R was replaced with both the YSR and CBCL in the current project.

##### Child behavior checklist (CBCL)

The emotional, behavior and somatic functioning, as well as social competence, were assessed by using the Arabic version of the CBCL questionnaires, licensed from the ASEBA/Research Center for Children, Youth & Families, University of Vermont, Burlington, VT USA. Of note, three statements about sexual problems were removed from the questionnaires due to cultural considerations.

CBCL is a validated questionnaire that assesses emotional and behavior problems in children age between 6 and 18 years [3, 22]. It consists of two main domains: 1) Problem Checklist, and 2) Social Competence. The Problem Checklist contains 112 statements which are grouped into three subscales; a) broad-band internalizing and externalizing problems, b) eight narrow-band syndromes, and c) Diagnostic and Statistical Manual of Mental Disorders (DSM)-oriented scales.

The second domain: the Social competence comprises seven statements that cover three areas; social relations, physical activities, and the mean of self-reported academic performance. Each statement is rated as 0 (not true), 1 (somewhat or sometimes true), or 2 (very true or often true).

#### Statistics

The proper data grouping into the subscales was performed by using the licensed software scoring program (ASEBA version 9.1). As a result, percentiles and T-scores are presented for all subscales and syndromes. The normal T-score range for all syndromes is 50–64, the borderline clinical range is 65–69, while the clinical range is 70–100. In regards to Social competence, the normal T-score range is 36–65, the borderline clinical range is 32–35, and the clinical range is 20–31.

The 386 participants were divided into three groups; non-TMD, TMD-pain and TMD-pain-free depending on the diagnoses presented in our previous study [10]. The mean and standard deviation (SD), median and (IQR) and also frequencies (%) are presented as descriptive statistics. To analyze differences in T-scores between the groups, the median score was modeled using quantile regression. In the unadjusted model TMD groups were included as dichotomous dummy variables with non-TMD pain as the reference group. The multivariate model adjusted for potential confounding factors included sex (male/female), age (10–13 years/14–18 years), and Saudi Arabian nationality (yes/no) as dichotomous variables. Also, the family income was modeled as dichotomous dummy variables that included three categories (below average/average/above average). Those categories were based on the average income in Saudi Arabia for the year 2013 (15,000 SR/month) (https://www.stats.gov.sa/). Subgroup analyses stratified by sex and age groups separately were performed with the non-TMD group as reference and with the stratification variable excluded from the model. *P*-values were based on 100 bootstrap samples. P-values lower than 0.05 and confidence intervals not including 0 were considered statistically significant. All analyzes were performed in STATA 12 SE.

## Results

### Demographic characteristics

Table 1 presents children and adolescents categorized into three groups; a) the non-TMD group; i.e. children and adolescents with no definite TMD diagnosis and no orofacial pain, b) the TMD-pain-free group; children and adolescents diagnosed with osteoarthrosis and/or disc displacement with or without reduction, and c) TMD-pain group; children and adolescents diagnosed with myofascial pain with or without limited mouth opening and/or arthralgia and/or osteoarthritis. As presented in our previous studies, there were no significant differences between the groups in regards to demographic characteristics [2, 10].

**Table 1:** Patient characteristics for 386 participants in Jeddah, Saudi Arabia

	Non-TMD n (%)	TMD-pain-free n (%)	TMD-pain n (%)
Individuals	279 (72.3)	22 (5.7)	85 (22)
Age (years)			
Mean (SD)	13.7 (2.2)	14.6 (2.3)	14 (2.2)
Median	13	14	13
Min-max	10–18	12–18	11–18
10–13 years	156 (55.9)	9 (40.9)	43 (50.6)
14–18 years	123 (44.1)	13 (59.1)	42 (49.4)
Sex			
Boys	101 (36.2)	9 (40.9)	25 (29.4)
Girls	178 (63.8)	13 (59.1)	60 (70.6)
Birth place			
Saudi Arabia	261 (93.5)	20 (90.9)	79 (92. 9)
Non-Saudi^a^	18 (6.5)	2 (9.1)	6 (7.1)
Nationality			
Saudi Arabia	177 (63.4)	10 (45.5)	49 (57.6)
Non-Saudi^a^	102 (36.6)	12 (54.5)	36 (42.4)
Family income			
Below average	152 (55.9)	11 (52.4)	46 (54.8)
Average	93 (34.2)	9 (42.9)	26 (31)
Above average	27 (9.9)	1 (4.8)	12 (14.3)

^a^Middle East, Gulf Area and Africa

All results presented below concerns the parents’ reports of their child’s status. To simplify reading, this has been omitted.

### Physical activities and social competence

The mean values of the children’s physical activities and social competence were within normal range for all three groups (Table 2). In this respect, there were no significant differences between the TMD-pain group and the TMD-pain-free group or the non-TMD group. When only the pain-free groups were compared, the adjusted and unadjusted analysis showed significantly higher ratings for physical activity in the TMD-pain-free group.

**Table 2:** The parents’ reports of children’s physical activities and social competence in 386 randomly selected children and adolescents in the general population of the city of Jeddah, Saudi Arabia

	Non-TMD	TMD-pain-free	TMD-pain
Activites (scale range)			
Mean (SD)	35.2 (8)	37.6 (9.4)	37.9 (8.8)
Median (IQR)	34 (44)	40 (36)	37 (41)
Min-max	20–64	20–56	20–61
Unadjusted	ref.	6*	3
95% CI		0.7–11.3	(−0.1)-6.1
Adjusted		5*	3
95% CI		0.8–9.2	(− 0.1)- 6.0
Social (scale range)			
Mean (SD)	40 (6.5)	40.8 (6.5)	40.5 (6.9)
Median (IQR)	39 (38)	41 (23)	39 (37)
Min-max	24–62	29–52	25–62
Unadjusted	ref.	2	0
95% CI		(−3.1)-7.1	(−2.6)-2.6
Adjusted		2	0
95% CI		(−2.7)-6.7	(−2.2)-2.2

The regression analysis of TMD-pain and TMD-pain-free were calculated with Non-TMD as reference group (ref.) in the quantile regression model

*significant association

When we stratified for sex, the adjusted analysis presented significantly higher scores for physical activities for boys in the TMD-pain-free group (Coefficient = 8; 95% CI: 2.0–14.0), but not for boys with TMD-pain (Coefficient = − 1; 95% CI: -5.7- 3.7).

Unadjusted analysis revealed significantly higher T-score for physical activities in the younger age group (10–13 years) with TMD-pain. Adjusted analysis showed significantly higher T-scores in the TMD-pain-free group (Coefficient = 5.5; 95% CI: 0.84–10.2) in the older age group (14–18 years).

In respect to social competence, unadjusted analysis revealed significantly higher T-scores in the TMD-pain-free group (Coefficient = 4; 95% CI: 0.20–7.8) than in the other groups, but this was not found in the adjusted analysis.

### Broadband internalizing and externalizing scale and narrow-band syndrome scale

For all narrow-band syndromes, the mean and median range of T-scores were within normal range in all three groups (50–64). In the unadjusted analysis, the TMD-pain-free group showed significantly higher T-scores for one externalizing problem (Rule-Breaking Behavior syndrome). Both the unadjusted and adjusted analyses showed higher T-scores for all internalizing problems and for one externalizing problem (Aggressive Behavior) in the TMD-pain group, as shown in Table 3. When we stratified for sex, girls in the TMD pain group showed significantly higher scores for Anxious/Depressed (Coefficient = 7; 95% CI: 3.0–11), Withdraw/Depressed (Coefficient = 3; 95% CI: 0.004–6.0), and Somatic complaints (Coefficient = 4; 95% CI: 0.7–7.3), in the unadjusted analyses. These findings remained almost the same in the adjusted analysis; Anxious/Depressed (Coefficient = 7; 95% CI: 3.8–10.2), Withdraw/Depressed (Coefficient = 3; 95% CI: -0.13-6.0, no longer significant), and Somatic complaints (Coefficient = 6; 95% CI: 3.0–9.0). Boys with TMD-pain showed significantly higher scores for somatic complaints in both the unadjusted (Coefficient = 11; 95% CI: 5.0–17.0) and adjusted analyses (Coefficient = 8; 95% CI: 2.8–13.2). Younger children with TMD-pain scored significantly higher for internalizing disorders in both the unadjusted and adjusted analyses; Anxious/Depressed (Coefficient = 6; 95% CI: 0.8–11 and Coefficient = 6; 95% CI: 1.5–10.5), Withdraw/Depressed (Coefficient = 3; 95% CI: -0.13-6.13 and Coefficient = 4; 95% CI: 0.9–7.1), and Somatic complaints (Coefficient = 6; 95% CI: 1.9–10.1 and Coefficient = 8; 95% CI: 4.5–11.5).

**Table 3:** Associations between TMD and eight narrow-band syndromes extracted from the child behavior checklist (CBCL)

	Syndromes	Non-TMD	TMD-pain-free	TMD-pain
Internalizing problems	Anxious/Depressed			
	Unadjusted Coeff.	ref	2	5*
	95% CI		(−2.3)-6.3	1.3–8.7
	Adjusted Coeff.	ref	2	6*
	95% CI		(−1.6)-5.6	1.8–10.2
	Withdrawn/Depressed			
	Unadjusted Coeff.	ref	-1	3*
	95% CI		(−5.7)-3.7	0.3–5.7
	Adjusted Coeff.	ref	−1.5	3.5*
	95% CI		(−5.1)-2.1	0.6–6.4
	Somatic Complaints			
	Unadjusted Coeff.	ref	0	5*
	95% CI		(−4)-4	(1.3)-8.7
	Adjusted Coeff.	ref	0	6*
	95% CI		(−2.8)-2.8	3–9.1
Externalizing problems	Social Problems			
	Unadjusted Coeff.	ref	0	2
	95% CI		(−4.4)-4.4	−1.7-5.7
	Adjusted Coeff.	ref	0	3
	95% CI		(−5.2)-5.2	(−0.6)-6.6
	Thought Problems			
	Unadjusted Coeff.	ref	−1	1
	95% CI		(−3.4)-1.4	(−2)-4
	Adjusted Coeff.	ref	−0.7	0.7
	95% CI		(−1.7)-0.4	(−2)-3.3
	Attention Problem			
	Unadjusted Coeff.	ref	0	1
	95% CI		(−2.9)-2.9	(−0.9)-3
	Adjusted Coeff.	ref	1	1
	95% CI		(−1.4)-3.4	(−0.7)-2.7
	Rule-Breaking Behavior			
	Unadjusted Coeff.	ref	3*	1
	95% CI		0.2–5.8	(−1.5)-3.5
	Adjusted Coeff.	ref	2.5	1
	95% CI		(−0.3)-5.3	(−1.1)-3.1
	Aggressive Behavior			
	Unadjusted Coeff.	ref	0	3*
	95% CI		(−2.7)-2.7	0.2–5.8
	Adjusted Coeff.	ref	0	4*
	95% CI		(−3.3)-3.3	1.3–6.7

The regression analysis of TMD-pain and TMD-pain-free were calculated with Non-TMD as reference group (ref.) in the quantile regression model

Unadjusted analysis and analysis adjusted for age, sex, ethnic origin and parental income is presented. Regression coefficients are presented with 95% confidence intervals retrieved from quantile regression analysis

*significant association

In regards to externalizing disorders, the score for rule breaking syndrome was significantly higher in girls with TMD-pain (Coefficient = 3; 95% CI: 0.5–5.5) Adjusted analysis showed higher score in the older age group with TMD-pain for rule breaking syndrome (Coefficient = 3; 95% CI: 0.2–5.8). Further, the score for aggressive syndrome was significantly higher in boys with TMD-pain in the adjusted analysis (Coefficient = 5; 95% CI: 0.13–9.9). Both the unadjusted and adjusted analysis showed significantly higher T-scores for younger children with TMD-pain for aggressive syndrome (Coefficient = 5; 95% CI: 1.4–8.6 and 5; 95% CI: 1.5–8.5, respectively).

### DSM-oriented scales

In unadjusted and adjusted analyses, the TMD-pain group differed significantly from the non-TMD group regarding affective, anxiety, and somatic problems in the DSM-oriented scales (Table 4).

**Table 4:** Associations between TMD and DSM-Oriented scale: Regression coefficients are presented with 95% confidence intervals retrieved from quantile regression analysis

	Non-TMD	TMD-pain-free	TMD-pain
Affective Problems			
Unadjusted Coeff.	ref	−1	5*
95% CI		(−4.5) − 2.5	(1.2)-8.8
Adjusted Coeff. ref		-2	4*
95% CI		(−6.2)-2.5	0.83–7.2
Anxiety Problems			
Unadjusted Coeff.	ref	0	5*
95% CI		(−5.7)-5.7	0.7–9.3
Adjusted Coeff. ref		0	5*
95% CI		(−6.0)-6.0	1.8–8.2
Somatic Problems			
Unadjusted Coeff.	ref	0	9*
95% CI		(−2.7)-2.7	4.6–13.4
Adjusted Coeff. ref		0	9*
95% CI		(−3.2)-3.2	4.8–13.2
Attention Deficit/Hyperactivity Problems			
Unadjusted Coeff.	ref	−1	0
95% CI		(−4.7)-2.7	(−3.4)-3.4
Adjusted Coeff. ref		0	1
95% CI		(−2.7)-2.7	(−2)-4
Oppositional Defiant Problems			
Unadjusted Coeff.	ref	0	1
95% CI		(−1)-1	(−0.5)-2.5
Adjusted Coeff. ref		0	1
95% CI		(−0.6)-0.6	(−0.4)-2.4
Conduct Problems			
Unadjusted Coeff.	ref	2	2
95% CI		(−2.2)-6.2	(−1.2)-5.2
Adjusted Coeff. ref		2	2
95% CI		(−3.4)-7.4	(−0.9)-4.9

The regression analysis of TMD-pain and TMD-pain-free were calculated with Non-TMD as reference group (ref.) in the quantile regression model

Both unadjusted analysis and adjusted for age, sex, ethnic origin and parental income are presented

*significant association

When the DSM-oriented scales were analyzed separately for age and sex, significantly higher Anxiety Problems in both the unadjusted and adjusted analysis (Coefficient = 6; 95% CI: 0.5–11.5 and Coefficient = 5.5; 95% CI: 1.1–9.8), Somatic Problems (Coefficient = 9; 95% CI: 4.4–13.6 and Coefficient = 6; 95% CI: 1.2–10.8) and Conduct Problems (Coefficient = 4; 95% CI: 0.5–7.5 and Coefficient = 4; 95% CI: 0.4–7.6) were found among girls with TMD-pain. Boys with TMD-pain showed significantly higher score in both the unadjusted and adjusted analysis for Somatic Problems (Coefficient = 9; 95% CI: 4.4–13.6 and Coefficient = 6; 95% CI: 1.2–10.8, respectively).

In the younger age group (10–13 years) TMD-pain was associated with significantly higher score in the unadjusted and adjusted analysis for Affective Problems (Coefficient = 6; 95% CI: 0.4–11.6 and Coefficient = 5; 95% CI: 1.0–9.0), Anxiety Problems (Coefficient = 5; 95% CI: 0.2–9.8 and Coefficient = 5.5; 95% CI: 1.1–9.8), Somatic Problems (Coefficient = 9; 95% CI: 4.4–13.6 and Coefficient = 5; 95% CI: 1.1–8.9) and Conduct Problems (Coefficient = 4; 95% CI: 0.5–7.5 and Coefficient = 3; 95% CI: 0.3–5.7) .

## Discussion

The main finding of the current study is that parents of children suffering from painful TMD conditions, as indicated by the CBCL, reported that their children have emotional problems, somatic problems, and aggressive behavior. Parents of children/adolescents with painful TMD conditions also reported that their children have internalizing problems, such as Anxious/Depressed, Withdrawn/Depressed, Somatic Complaints, in contrast to parents of children/adolescents with non-painful TMD conditions. These findings are not surprising and coincide well with the results from our previous study assessing the same psychometric variables but from the children/adolescents themselves using YSR [2]. Also, other studies using CBCL to evaluate psychometric variables of children and adolescents suffering from pain due to different health conditions have shown similar findings in this study. Those studies reported that parents rated children complaining of pain with a higher degree internalizing problems such as withdrawn, somatic complaints, and anxious/depressed, than parents of children with no pain [4, 6, 7, 23]. Among those studies, one study highlighted the importance of multidimensional assessment models, such as CBCL, to be used for children with chronic musculoskeletal pain [23].

In regards to externalizing problems, the parents of children and adolescents who suffered from TMD-pain rated the children and adolescents as aggressive. Similar results were reported by children and adolescents, who suffered from TMD-pain when they asked about their emotional functioning in our previous study [2]. List et al. (2001) found the aggressive behavior among adolescents with TMD-pain, when children-rating scale (YSR) were used, compared to healthy controls [15]. Unlike the current study, our previous study showed that social as well as thought problems were associated with children and adolescents having TMD-related pain [2]. However, the parents in the current study did not indicate that they were aware of their children social and thought problems. An explanation for this difference is that YSR is a child-rating scale that subjectively measures the child’s real perception regarding their feelings. Whereas, the parent rating scale (CBCL) was shown to efficiently measure the externalizing problems in their offspring rather than internalizing problems [24].

While the DSM-oriented scale was analyzed in the current study, the parents of children and adolescents in the TMD-pain group revealed that their children complain of anxiety, affective, and somatic problems. This finding ascertained similar results with the previous study from our group, in which children and adolescents with TMD-pain reported that they suffered from anxiety, affective, and somatic problems as well [2]. This indicates that parents are aware of their children’s problems and therefore they can help their children to manage those problems early in life. This recommendation is to prevent the emotional and behavioral problems that meet the criteria of DSM-VI from being sustained to adulthood as suggested in one longitudinal study [25]. One explanation for the continuation of the emotional and behavioral problems into the adulthood is that TMD-pain and its associated emotional problems share memories from early pain experiences, then it is easy to recall these associations later in life [18, 26]. With respect to physical activities the parents of children and adolescents with non-painful TMD rated that their children were reasonably physically active. This finding is a contrast to the findings from our previous study in which children and adolescents in the TMD-pain-free group reported a lower rate of sports activities [2] and might confirm the importance of using self-report measures among youth who suffer from different pain conditions.

Consistent with our previous study, the parents’ report revealed that social relations were within the normal range in all groups [2]. However, parents indicated that the risk of having depressive and somatic symptoms was higher among girls than boys with TMD-pain. Although this finding is in contrast to our previous study, studies indicate that girls with TMD-pain report higher degree of depressive and somatic problems than boys [1, 15]. Further, the parents’ report that their girls with TMD-pain possess rule breaking behavior to a higher degree than boys with TMD-pain, while aggressive behavior is more common in boys with TMD-pain. However, these associations were not found in our previous study when the child-self rating scale was used. This difference in the findings between our two studies can be explained by the notion that the YSR is subjectively measuring the perception of the child’s own behavior, while CBCL measures the parent opinion about their off-spring. Nevertheless, many other studies showed that parents reported fewer or more problems than their children/adolescents do [27–29]. This might indicate the importance of using the YSR in evaluating the emotional and behavior problems especially for adolescents with painful TMD conditions. The YSR may therefore be recommended in children that are mature enough to include all possible internalizing and externalizing problems precisely, especially during diagnosing, whereas the CBCL seems useful in young children with painful TMD conditions. Especially in children that are too young to fill in a questionnaire by themselves. With this in mind, and the fact that children and adolescents are frequently experiencing emotional and behavioral consequences due to pain [2], one has to consider if there is a need to also have the parents’ view point (using for instance the CBCL). Also, since it has been shown that a comorbidity with psychiatric conditions and psychological distress has a significant and negative outcome for patients with chronic conditions like headache [16], one can assume that the CBCL also could be used as reliable predictor for the outcome of TMD.

One of the strengths of the current study is that the one examiner performed the RDC/TMD examination. This examiner was trained and calibrated with a gold standard clinician who is specialized in Orofacial Pain and Jaw Function. Another strength is the use of a reliable examination method (RDC/TMD) for children and adolescents and also a validated questionnaire, which has been used in many previous studies among children [11, 30, 31]. The random enrollment of participants is another strength.

One limitation of the current study could be the high drop-out rate among boys. Despite the settings for boys and girls were equal, the higher drop-out rate among boys was difficult to avoid as the place of examination were not the same among boys and girls. While the boys were invited to visit the nearest primary health care center, the girls were examined in the nurse room inside each school. A second reason to the higher drop-out rate in boys could be that Saudi girls are showing more dental care awareness than boys [32]. Another limitation of the study is the lack of information on the parents answering, i.e. sex, age, educational level, income, etc. Thus, we cannot be certain that the children and parents understood and interpreted the questions correctly.

## Conclusion

In conclusion, the present study revealed that the parents rated that their children with TMD-pain suffer from emotional, somatic and aggressive behavior to a higher degree than healthy control subjects. The main outcome from the present study emphasizes the importance of using self-reported measures among youth suffering from pain conditions. For children that are too young to fill in a questionnaire by themselves parent-reported scales are suggested.

## Abbreviations

CBCL: Child Behaviour Checklist
RDC/TMD: Research Diagnostic Criteria for Temporomandibular disorders
TMD: Temporomandibular disorders
TMJ: Temporomandibular joint
YSR: Youth Self Report Scale
